# Quantitative Aspects of Glow Discharge Mass Spectrometry

**DOI:** 10.6028/jres.093.104

**Published:** 1988-06-01

**Authors:** N. E. Sanderson, P. Charalambous, D. J. Hall, R. Brown

**Affiliations:** VG Elemental, Ion Path, Road Three, Winsford, Cheshire, England

The problems of quantitation that arise in the direct analysis of solid samples are well known. Amongst them are the preparation and certification of homogeneous standards, and the transfer of calibration factors from standards to similar or dissimilar matrices. These problems have been more aggravated as a result of the increased need for trace analysis at sub-ppm (microgram/gram) levels. Laboratory based instruments traditionally used in this area suffer from poor precision or large matrix effects.

The glow discharge mass spectrometer (GDMS) offers some attractive possibilities in this regard. The glow discharge source delivers a stable, high intensity, ion beam enabling good precision to be achieved from the electrical detection system. The method of ion formation in the source involves sputtering neutral atoms from the sample and then ionizing them in the plasma. This two-step process is expected to considerably reduce dependence on matrix effects, giving relative ion yields that are not very sensitive to the material or chemical compositions of the sample. Finally, the application of high resolution mass spectrometry removes spectral interferences which in many cases would cause problems for elemental signals at ppm or lower. Results have been obtained by a number of laboratories operating GDMS (VG 9000—manufactured by VG Elemental), which show the above features. Table 1 is a compilation [1] of relative ion yields obtained from a range of standards of different materials. They have been normalized to the value for iron, and it can be seen that the relative yields vary by less than 30% for these dissimilar matrices.

The relative ion yields in table 1 can be used to construct a table of average relative sensitivity factors which can then be applied to matrices for which no convenient standard exists. The expectation is that, in general, this should provide quantitative analysis to within 30%, which is often sufficient at trace levels. Table 2 shows a test of this using a molybdenum sample [2]. After correction with average factors the results are well within 30% of the certified values. Other examples have been obtained. Quantitation can be further improved by using appropriate standards under controlled conditions. In these cases accuracies of 1–2% have been achieved.

Good internal and external precisions are inherent in achieving good quantitation. However, such measurements inextricably link sample homogeneity and instrumental reproducibility. Table 3 shows precisions [2] measured using a VG 9000 on 10 separate samples taken from a block of aluminum. Internal/external precisions of a few percent at the ppm level and 10–20% at the tens of ppb level show *both* good sample homogeneity and instrumental reproducibility. This exercise was in fact undertaken to assess the homogeneity of the material prior to a round-robin exercise.

## Figures and Tables

**Table 1:** Relative ion yields for various matrices with the VG 9000 GDMS

Element	Ga	Al	Fe	Cu	Mean RIY	Max deviation %
B		1	1	1.2	1.1	9
Na	1	0.5	0.66		0.72	38
Mg	1	.77	.91		.89	13
Al	0.85	1	.63	1	.87	27
Si	.63	0.91	.5	1	.76	31
S	.5			0.34	.42	19
Cr	.5	.5	.5	.8	.6	33
F	1	1	1	1	1	
Cu	0.34	0.25	0.25	0.30	0.29	17
Ga	.5	.4			.45	11
Zn	.15	.2		.15	.16	25
Se	.3			.33	.34	3
Ge	.63		.5		.56	12
Cd	.18	.17		.17	.17	6
Sn	.34	.4	.42	.43	.42	2
Te	.34			.36	.35	3
Pb	.36	.34	.34	.38	.36	5

**Table 2:** Analysis of molybdenum sample following calibration with average sensitivity factors

Element	VG 9000 result using average sensitivity factors (ppm wt)	Quoted conc. (ppm wt)
Co	24	21
Cr	51	45
Cu	33	28
Fe	2125	1880
Mn	19	18
Ni	29	28
Si	151	133
V	1.4	1
w	5075	4700
Zr	99	120

**Table 3:** VG 9000 analysis of BCR high purity aluminum

Element	Conc. ppm (ion beam ratio)	RSD %
	0.800^a^	10
Mg	.792^b^	8.5
	.755	6.3
Si	.766	2.5
	.126	19
Ca	.149	15.4
	.272	11.8
Ti	.243	14.4
	.048	6.3
V	.047	6.4
	.010	20
Cr	.011	20
	.036	8.3
Mn	.038	7.8
	.497	10
Fe	.519	5.6
	.042	26
Ni	.054	14.8
	.130	42
Cu	.128	27.3
	.017	23.5
Zn	.019	10.5
	.017	11.8
Pb	.017	11.8
	.007	14.2
Th	.006	14.6
	.005	20
U	.005	20

a Upper level figures are internal measurements (same sample).

b Lower level figures are external measurements (different samples).
